# Tracking ocean heat uptake during the surface warming hiatus

**DOI:** 10.1038/ncomms10926

**Published:** 2016-03-30

**Authors:** Wei Liu, Shang-Ping Xie, Jian Lu

**Affiliations:** 1 Climate, Atmospheric Sciences, and Physical Oceanography (CASPO), Scripps Institution of Oceanography, University of California, San Diego, 9500 Gilman Drive, La Jolla, California 92093, USA; 2 Pacific Northwest National Laboratory, 902 Battelle Blvd, Richland, Washington 99354, USA

## Abstract

Ocean heat uptake is observed to penetrate deep into the Atlantic and Southern Oceans during the recent hiatus of global warming. Here we show that the deep heat penetration in these two basins is not unique to the hiatus but is characteristic of anthropogenic warming and merely reflects the depth of the mean meridional overturning circulation in the basin. We find, however, that heat redistribution in the upper 350 m between the Pacific and Indian Oceans is closely tied to the surface warming hiatus. The Indian Ocean shows an anomalous warming below 50 m during hiatus events due to an enhanced heat transport by the Indonesian throughflow in response to the intensified trade winds in the equatorial Pacific. Thus, the Pacific and Indian Oceans are the key regions to track ocean heat uptake during the surface warming hiatus.

## Abstract (web summary)

Mechanisms for the warming hiatus are inferred by tracking regional ocean heat uptake in different regions. Here, the authors show that the Indo-Pacific heat redistribution holds the key while the abyssal heat uptake in the Atlantic and Southern Oceans primarily reflects the downward penetration of anthropogenic heat.

## Graphical abstract

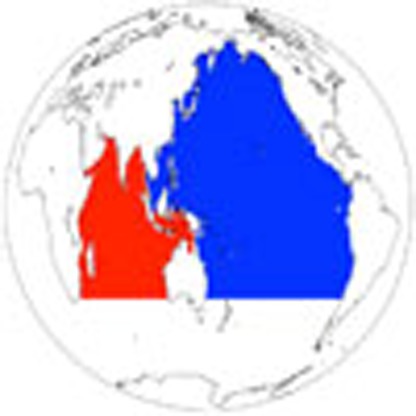

The global surface temperature increase has slowed over the past 16 years^1^,^2^,^3^. The warming hiatus is regarded as a result of natural variability against the centennial warming trend, while radiative forcing change also contributes^4^,^5^,^6^. Two leading theories have been proposed to explain the hiatus. One suggests that the hiatus is closely associated with the negative phase of the Interdecadal Pacific Oscillation (IPO)^7^,^8^,^9^, manifested as a La Niña-like cooling and intensified easterly winds over the equatorial Pacific^10^,^11^. Consistent with this theory, the recent hiatus can be successfully simulated by nudging the sea surface temperature (SST) or trade winds to observations over the tropical Pacific^12^,^13^,^14^. The other idea is based on an energy view, focusing on the vertical energy redistribution in the ocean on the decadal timescale. The surface warming hiatus is accompanied by an excessive global deep ocean (>700 m) heat uptake^15^, consistent with a positive radiative imbalance at the top of the atmosphere^16^,^17^,^18^,^19^,^20^. These two theories are not mutually exclusive. Indeed, the accelerated trade winds at the IPO-negative phase tilts the equatorial thermocline, causing an apparent vertical heat redistribution^13^,^21^.

Recent studies^21^,^22^,^23^ further attempted to infer mechanisms for the warming hiatus by tracking regional ocean heat uptake at different levels. Specifically, an earlier model study^21^ found that surface warming hiatus is significantly correlated with accelerated warming in deep levels in all basins, below 750 m in the Atlantic and Southern Oceans and below 300 m in the Pacific and Indian Oceans. Observational data analyses, however, showed different results, from models and between data sets. In the Ishii data (Methods), the increase of ocean heat content (OHC) between 300 and 1,500 m accelerates in the Atlantic and Southern Oceans but changes little in the Pacific and Indian Oceans^22^. In the World Ocean Atlas (WOA) data (Methods), on the other hand, the recent surface warming hiatus is associated with a heat rearrangement within the upper 300 m between the Pacific and Indian Oceans^23^, along with an increased heat transport from the Pacific to the Indian Ocean by the Indonesian throughflow (ITF)^24^. These debates highlight a gap between models and observations, large uncertainties in OHC observations^19^,^25^,^26^,^27^,^28^, and a complex relationship between global mean surface temperature (GMST) and OHC^29^.

In this study, we revisit the energy theory and combine multiple observational data sets and state-of-art model simulations to examine the relationship between the GMST and regional OHC on decadal timescales. We show that the deep ocean heat uptake in the Atlantic and Southern Oceans primarily reflects the downward penetration of anthropogenic heat via the deep meridional overturning circulation (MOC), while OHC in the upper 350 m in the Pacific and Indian Oceans is closely tied to the surface warming hiatus.

## Results

### Global and regional OHC changes

**Figure 1:**
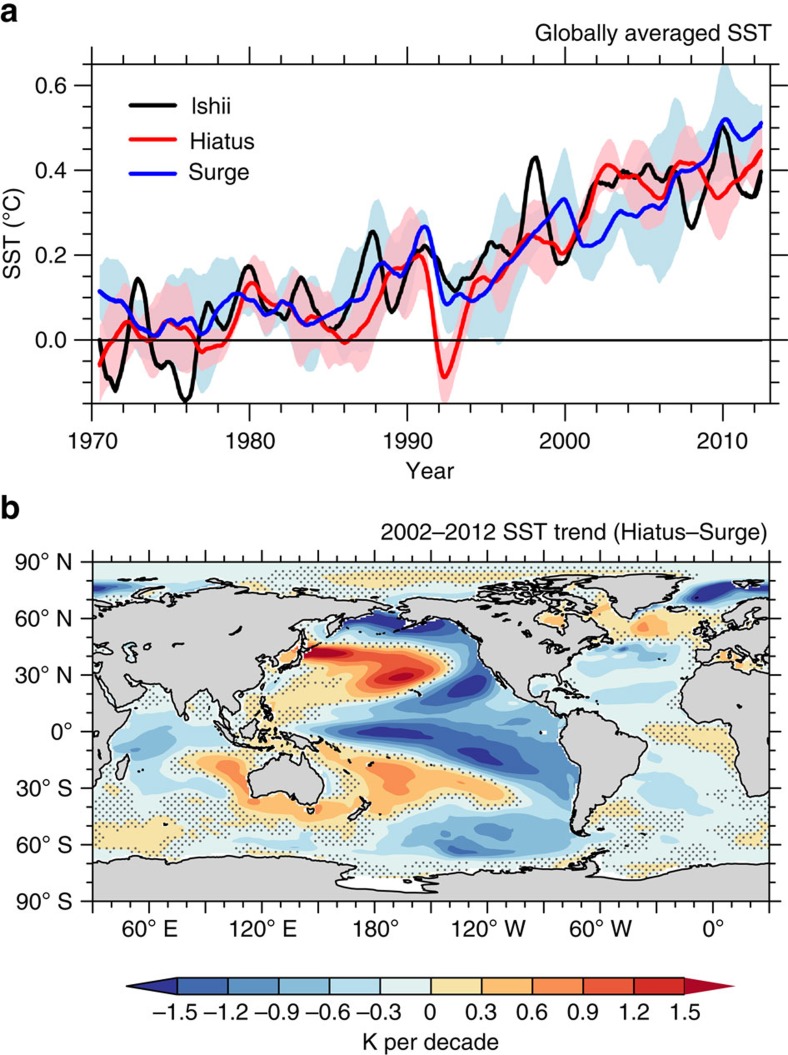
Observed and simulated SST. (**a**) Monthly mean globally averaged SST from the Ishii data (black) and the CESM ensemble simulations. Two groups (named the Hiatus and Surge) of ensembles are shown, whose variations (one s.d.) are shaded in light pink and light blue and ensemble means are drawn in red and blue. All the curves are shown as a 12-month running mean by subtracting the annual mean value of the first year (year 1970). (**b**) The SST trend difference between the ensemble means of the CESM Hiatus and Surge groups during 2002–2012 (shading in *K* per decade). Stippling indicates region below 95% significance computed from a two-tailed *t*-test.

**Figure 2:**
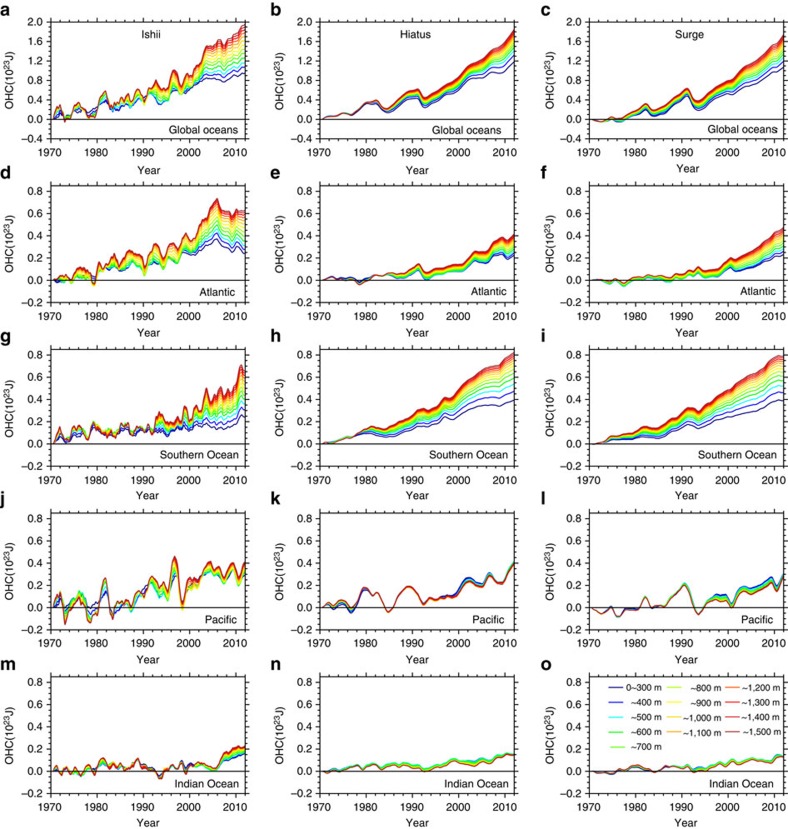
Observed and simulated OHC. OHC integrated from the surface to indicated depths in global oceans, the Atlantic, Southern Ocean, Pacific and Indian Ocean from the Ishii data (**a**,**d**,**g**,**j,m**) and the ensemble means of the CESM Hiatus (**b**,**e**,**h**,**k**,**n**) and Surge (**c**,**f**,**i**,**l**,**o**) groups. All the curves are shown as a 12-month running mean by subtracting the annual mean value of the first year (year 1970).

The observed GMST (defined as globally averaged SST in this study, not including land surface temperature) from the Ishii data^30^ (Methods) shows a surface warming slowdown since 1998, with a near-zero warming trend during 2002–2012 (Fig. 1). Using the same data, ref. 22 shows that the OHC anomalies (from the 1970–2012 mean) increase during the hiatus period, and the warming penetrates deeper than 300 m only in the Atlantic and Southern Oceans (Supplementary Fig. 1). The large heat penetration was then taken as suggesting the importance of these basins for the hiatus. Here, instead of referencing to the 1970–2012 mean as in ref. 22, we compute ocean temperature anomalies as deviations from the 1970 values because referencing to 1970 facilitates an easy inspection of the OHC variation over the entire analysis period. From our rendering in Fig. 2, the most prominent feature of historical OHC variation is the signal of anthropologic warming: both global and regional OHCs continuously increase during 1970–2012. The interbasin difference of OHC increase is not unique to the hiatus period but common to the entire analysis period. Between 300 and 1,500 m, the OHC in the Atlantic, Southern Ocean, Pacific and Indian Ocean (Supplementary Fig. 1) exhibits a warming trend of 0.19, 0.23, 0.05 and 0.09 × 10^23^ J per decade during 1998–2012, which accounts for 33.8%, 40.7%, 8.2% and 15.8% of the global OHC warming trend. Such interbasin partition holds when we extend the analysis period to 1970–2012 (and to other observational data sets, see Supplementary Fig. 2). The Atlantic, Southern Ocean, Pacific and Indian Ocean explain 30.7%, 41.3%, 13.5% and 5.4% of the global warming within 300–1,500 m, respectively. This result demonstrates the dominance of the Atlantic and Southern Oceans in the deep ocean sequestration of heat, and more importantly, indicates that the penetration of heat below 700 m is not uniquely tied to interdecadal modulations of the surface warming rate.

Model simulations are consistent with the above result. From the Community Earth System Model (CESM) large ensemble simulations^31^ (Methods), four members are selected each for the Hiatus and Surge groups. The Hiatus group members are where the decadal trend of GMST is once negative during 2002–2012, whereas the Surge group are the runs corresponding to individual Hiatus members but with the largest warming trend during the same span (Supplementary Fig. 3; Supplementary Table 1). In Fig. 1a, the Hiatus group features a warming hiatus, with a near-zero warming rate since the early 2000s, while the Surge group shows a continuous surface warming, much like the ensemble mean of the Couple Model Intercomparison Project phase 5 (CMIP5) models^8^,^32^. The Hiatus and Surge groups share the same radiative forcing, but have different initial conditions. The difference between two groups is thus due to natural variability. The spatial pattern of the Hiatus minus Surge difference is characterized by negative SST trends in the tropical Pacific, and positive trends in the subtropical North and South Pacific (Fig. 1b), a pattern resembling the La Niña-like negative phase of the IPO.

Despite the differences in the GMST warming rate, subsurface OHCs (Methods) in the Ishii data, the Hiatus and Surge groups share a common evolution pattern. During 1970–2012, the global OHC keeps increasing, and the rate increases when it is integrated over a greater depth (Fig. 2). The Atlantic and Southern Oceans contribute most to OHC increase at depths >300 m, and this dominance in deep ocean contribution is not dependent on the GMST warming rate. During 1998–2012, the increase of 300–1,500 m OHC in the Atlantic and Southern Oceans accounts, respectively, for 30.7% and 50.1% of global OHC increase in the Hiatus group, and 43.2% and 46.3% in the Surge group. It is clear that results from either group are consistent with observations that show a deep ocean uptake in these basins. The deep heat storage takes place in the Atlantic and Southern Oceans over the entire 43 years, regardless whether the GMST warming slows down (in the Ishii data and the Hiatus group) or accelerates (in the Surge group). The deep heat penetration in the Atlantic and Southern Oceans is the result of anthropogenic warming, not unique to the hiatus. Therefore, the deep warming in these two basins is not the basis to argue for their importance in driving the hiatus.

To further test the robustness of above results, we look for more warming hiatus and surge periods in the future events of the large ensembles. Running 10-year linear trends of GMST from 38-member simulations reveal another five hiatus events with negative decadal trends after 2012. There are nine hiatus decades in total. For each hiatus decade, we inspect the trends in the other 37 members and choose the one with the largest warming trend as the corresponding warming surge decade. The same span ensures the pair of hiatus and surge events share the identical radiative forcing. Thus, we expand the Hiatus and Surge groups to nine members each by including five pairs of hiatus (Supplementary Fig. 4) and surge decades (Supplementary Table 1) in the future simulations (the new groups are referred as the fHiatus and fSurge, see Methods). The decadal trend of composite GMST is -0.014±0.023 K per decade in the fHiatus group and 0.376±0.098 K per decade in the fSurge group, where the range (± one s.d. of group × 1.86) represents the 95% range from a one-sided Student's *t*-test^21^. The trend spreads of two groups do not overlap, so these two groups are well separated regarding the surface warming rate.

**Figure 3:**
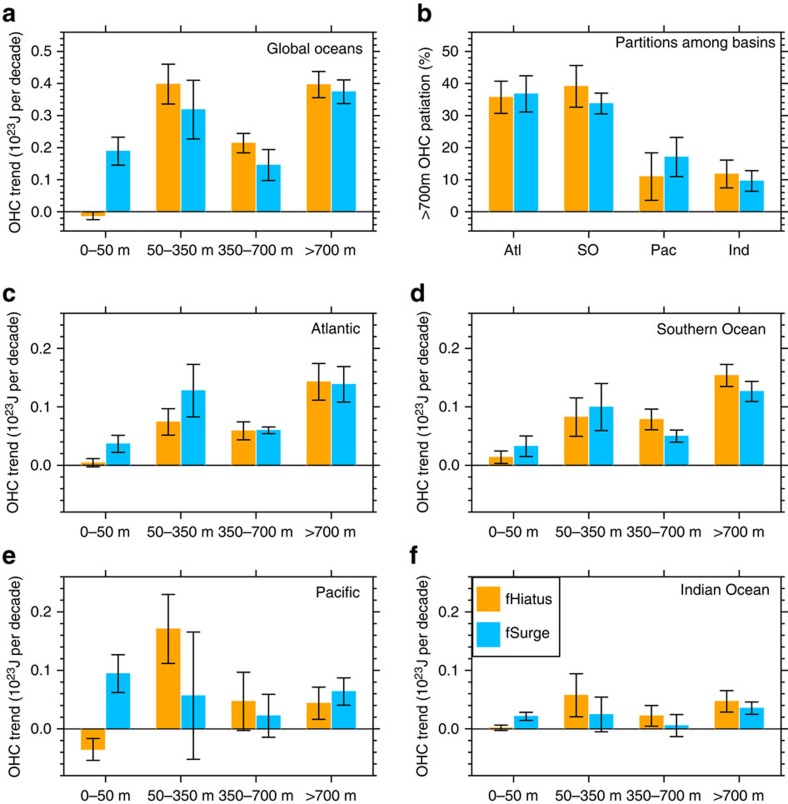
OHC during hiatus and surge events. (**a**) Composite linear trends of global OHC during hiatus (orange bars) and surge (deep sky-blue bars) decades in the 21st century for four layers (surface to 50 m, 50–350 m, 350–700 m and below 700 m) with error bars showing one s.d. (**c**–**f**) Similar to **a** but for the Atlantic, Southern Ocean, Pacific and Indian Ocean. (**b**) The partition of individual basins for the OHC trend in oceans deeper than 700 m.

Figure 3 compares the composite average trends of global and regional OHCs between the fHiatus and fSurge groups, where the error bar denotes one s.d. among members. To track the vertical heat redistribution over decades, we separate the ocean into four layers: 0–50 m, 50–350 m, 350–700 m and below 700 m. For the upper 350 m, the composite global OHC trend for nine hiatus decades is 0.39 × 10^23^J per decade, a reduction of 31.6% compared with the composite trend in the fSurge group. This reduction is compensated in the deeper layers where the OHC trend for the hiatus decades is greater than the surge decades. For the 350–700 m layer, the composite global average in the fHiatus group is 32.0% larger (0.21 versus 0.15 × 10^23^J per decade), and for the layer below 700 m, the composite global average in the fHiatus group is 5.6% larger (0.40 versus 0.37 × 10^23^J per decade). The vertical heat redistribution is such that more heat is stored in the deep layers during hiatus decades^21^.

Deep ocean below 700 m contributes ∼38% of the whole ocean heat uptake. The difference in deep ocean heat uptake between the hiatus and surge groups is one order of magnitude smaller than their mean uptake (Fig. 3a). The s.d. within each group is also a few times smaller than the group mean. Thus, the deep ocean heat uptake observed during the hiatus decade reflects primarily anthropogenic warming, not the decadal variations that cause the hiatus. In support of observational results^23^, our analyses of the large ensemble simulations show that statistically we do not expect to see a significant correspondence between decadal modulations of the GMST trend and global deep ocean heat uptake.

A clear pattern emerges from the regional ocean heat uptake below 700 m (Fig. 3b): it is large in the Atlantic and Southern Oceans (each making up ∼36% of the global uptake) but small in the Pacific and Indian Oceans (∼28% combined), a result consistent with observations^22^. Like the global mean, the deep ocean heat uptake in the Atlantic and Southern Oceans is dominated by anthropogenic warming, while the decadal variability (including the fHiatus-fSurge difference) is an order of magnitude smaller. We can therefore apply the same conclusion made for global heat uptake below 700 m to these individual basins: the penetration of heat below 700 m in these basins does not make a major contribution to surface hiatus events or surges, either individually, or collectively.

### Mechanisms for regional OHC variations

**Figure 4:**
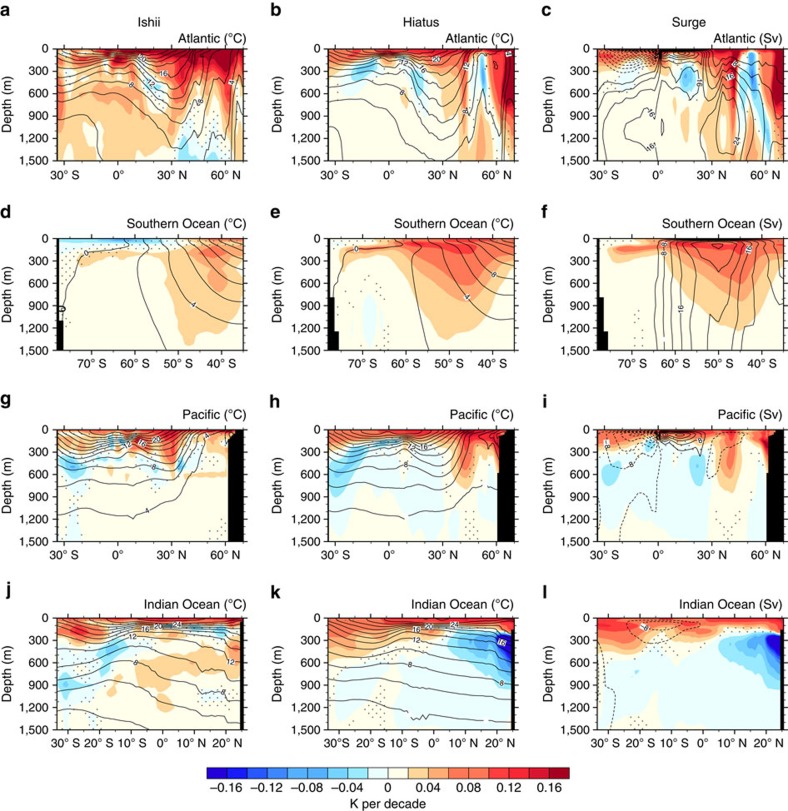
Observed and simulated subsurface temperature trends. Zonal mean temperature trends during 1970–2012 (shading in *K* per decade) in the upper 1,500 m in global oceans, the Atlantic, Southern Ocean, Pacific and Indian Ocean from the Ishii data (**a**,**d**,**g**,**j**) and the ensemble means of the CESM Hiatus (**b**,**e**,**h**,**k**) and Surge (**c**,**f**,**i**,**l**) groups. Stippling indicates region below 95% significance computed from a two-tailed *t*-test. Contours show the 1970–2012 climatology of isotherm (°C) and meridional overturning stream-function (*S*_*v*_, 1*S*_*v*_=1 × 10^6^ m^3^ s^−1^). Since isotherms and meridional overturning stream-functions are highly similar between the Hiatus and Surge groups, for simplicity, the former is included in panels of the Ishii data and the hiatus group, while the latter is included in panels of the Surge group.

Physically, the disconnection between the decadal variations in GMST and OHC below 700 m reflects the fact that the variations of GMST and OHC below 700 m are governed by different mechanisms. GMST is affected by the atmosphere–ocean interaction and SST variability is organized into coherent patterns like the IPO^8^,^9^,^13^. Below 700 m, temperature variations are governed by distinct subsurface ocean dynamics, especially the MOCs in the Atlantic and Southern Oceans. MOCs prove to set the depth at which anthropogenic warming penetrates, which extend to great depths (>1,500 m) in the Atlantic and Southern Oceans. The former is known as the Atlantic meridional overturning circulation (AMOC) (Fig. 4c) and the latter is a residual circulation resulting from wind-driven and eddy-mediating mechanisms (Fig. 4f). The sinking motions of the deep MOCs (40–80°N in the Atlantic and 40–50°S in the Southern Ocean) sequester anthropogenic heat in the deep ocean below 700 m in these two regions, causing a pronounced abyssal warming (Fig. 4a–f). MOCs in the Pacific and Indian Oceans, however, are limited to shallow depths (<∼300 m). They appear as symmetric cells about the equator in the former while as a single anticlockwise cell in the latter, trapping anthropogenic heat mainly within the upper ∼300 m in these two basins (Fig. 4g–l).

**Figure 5:**
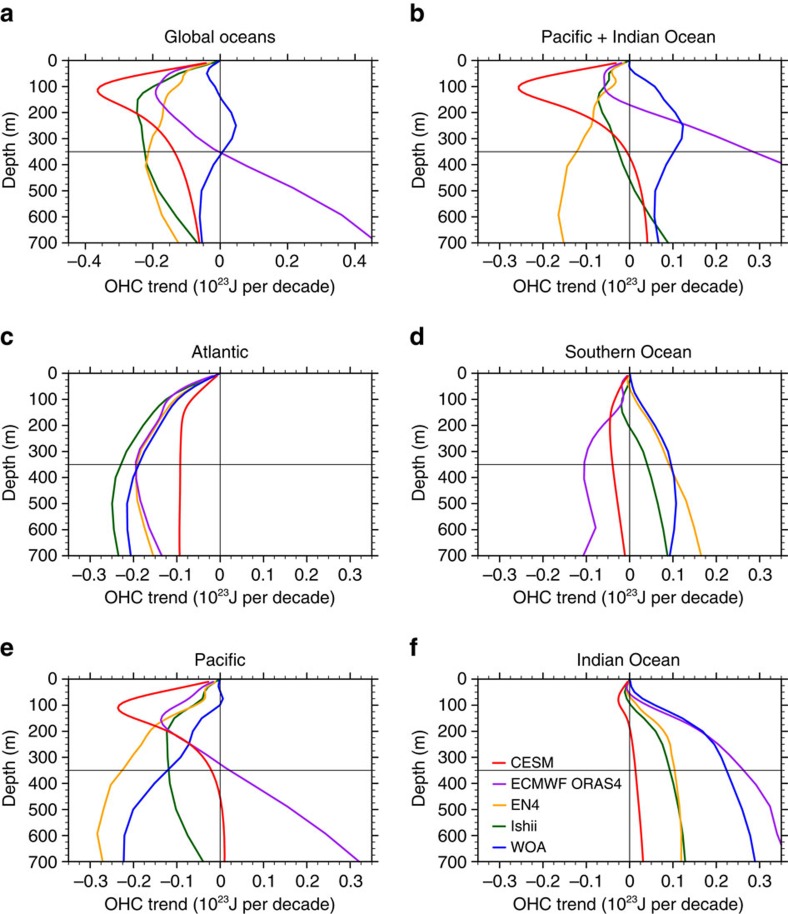
Global and regional OHC trend differences as a function of depth between Hiatus and Surge events. Results in (**a**) global oceans, (**b**) the Pacific and Indian Oceans, (**c**) the Atlantic, (**d**) the Southern Ocean, (**e**) the Pacific and (**f**) the Indian Ocean are from observational data sets and the CESM simulations. OHC is integrated from the surface to indicated depths. Observations include the ECMWF ORAS4 reanalysis product (purple), the EN4 data (orange), the Ishii data (dark green) and the WOA data (blue). The CESM result (red) shows the OHC trend difference between the fHiatus and fSurge groups (the fHiatus ensemble mean minus the fSurge ensemble mean).

Heat uptake above 700 m, by contrast, shows certain correlation with GMST. Within the top three layers, differences in Indo-Pacific heat uptake between the fHiatus and fSurge groups are robust and on the same order of magnitude as their mean uptakes (Fig. 3e,f). During hiatus periods, anomalous cooling happens in the surface mixed layer (0–50 m) in all basins, especially in the Pacific. Over the Pacific and Indian Oceans, the 0–50 m cooling is compensated by warming within 50–350 m (Fig. 3e,f). This result is different from an earlier model study^21^, which showed an overall cooling in the upper 300 m in the Indian Ocean but consistent with observations^23^. Here we include four observational data sets to compare with model results: the Ishii data, the EN4 data^33^, the WOA data^34^ and the latest European Centre for Medium-Range Weather Forecasts ocean reanalysis system 4 (ECMWF ORAS4) product^15^,^35^ (Methods). All the observational data sets and CESM simulation consistently show an anomalous warming in the Indian Ocean below ∼50 m (Supplementary Fig. 5e) and an accelerated OHC increase (Fig. 5f) during hiatus periods. This Indian Ocean OHC increase corresponds to a concurrent Pacific OHC decrease in the 0–100 m layer (Fig. 5e), indicating heat redistribution between these two basins. The distribution patterns vary among data sets due to data uncertainties, while the model result well lies within the range of observational uncertainties (Fig. 5b). Our model result shows that the total Indo-Pacific OHC change is close to zero within the upper 350 m (Fig. 5b), meaning that most of the hiatus-related cooling in the surface and mixed layer is compensated by warming in 50–350 m. Therefore, enhanced heat uptake below 350 m, as suggested by ref. 21, is not required in these two basins.

**Figure 6:**
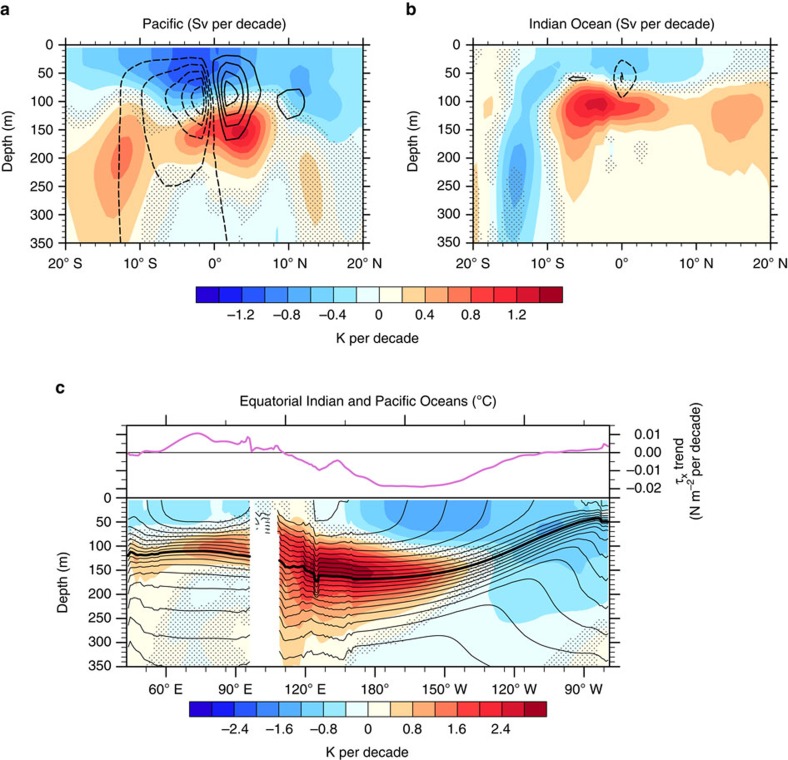
Temperature trend differences in the tropical Pacific and Indian Oceans in the upper 350 m between the CESM Hiatus and Surge groups during 2002–2012. (**a**) The trend difference of zonal mean temperature (shading in *K* per decade) in the tropical Pacific, superposed by the trend difference of meridional overturning stream-function (contoured by 2*S*_*v*_ per decade, with zero contours omitted). (**b**) Similar to **a** but for the tropical Indian Ocean. (**c**) Trend differences of zonal wind stress (orchid) and temperature (shading in *K* per decade) along equatorial band (5° S–5° N) in the Indian and Pacific Oceans. The mean isotherms during 2002–2012 (38-ensemble mean) are also included as contours with an interval of 1 °C. The 20 °C contour is thickened to indicate the depth of thermocline.

Figure 6b,c and Supplementary Fig. 6d further show that warming in the Indian Ocean mostly happens in the tropics at the thermocline depth (70–150 m). This warming pattern appears related to a shift towards a La Niña-like state^23^ and a change of the ITF^24^. To further investigate the La Niña-like shift and related Indo-Pacific heat rearrangement, we consider the difference of climate trend between the Hiatus and Surge groups during 2002–2012. Our results show a pattern in the Pacific consistent with the La Niña-like negative phase of the IPO as described by ref. 13. Specially, anomalous high sea level pressure (SLP) centres in the mid-latitudes and intensified trade winds reflect the accelerated Walker and Hadley cells (Supplementary Fig. 7a). Strengthened surface winds accelerate equatorial surface currents (Supplementary Fig. 7c), the Equatorial Undercurrent (EUC) (Supplementary Fig. 7d) and the Pacific shallow MOC (Fig. 6a and Supplementary Fig. 7e,f). The tropical thermocline deepens in the central and western Pacific (Fig. 6c; Supplementary Fig. 7b) with an anomalous subsurface warming maximum due to increased equatorial pycnocline heat convergence, and shoals in the eastern Pacific with enhanced upwelling and surface cooling (Figs 6c; 1b).

The anomalous subsurface warming and accelerated OHC increase in the Indian Ocean is closely associated with the change in the Pacific. Anomalous warm water in the tropical western Pacific can be transported into the Indian Ocean via the Indonesian passages. During hiatus events, both the ITF volume transport and the ITF heat transport markedly increase^24^, especially over the upper 350 m (Supplementary Fig. 8), which substantially contributes to the OHC increase in the Indian Ocean. Besides, local response in the Indian Ocean during the La Niña-like shift also facilitates the acceleration of the OHC increase in the Indian Ocean. Anomalous low SLP occurs in the subtropical southeastern Indian Ocean, with relaxed southeasterlies along Sumatra (Supplementary Fig. 7a). The weakened along-shore winds reduce the upwelling and deepen the thermocline off the coast. At the equator, anomalous westerlies pile up water eastwards and depress the thermocline in the eastern tropical Indian Ocean (Fig. 6c). Both processes contribute to the anomalous subsurface warming and OHC increase in the Indian Ocean.

## Discussion

We show that the observed global OHC in the 300–1,500 m is dominated by a continuous warming trend over the past 43 years, and the penetration depth of ocean warming in individual basins is determined by the depth of the regional MOC. The deep (700–1,500 m) ocean heat uptake observed during the 21st century in the Atlantic and Southern Oceans primarily reflects anthropogenic heat entering the subsurface ocean via the deep MOC. This deep penetration occurs at a similar rate during both hiatus and surge periods and is therefore not a major contributor to decadal changes in the warming rate of GMST. Our analyses of the CESM large ensemble simulations further show that the regional pattern of decadal ocean heat uptake below 700 m is indeed robust but corresponds poorly to decadal variations in global surface warming rate. OHC in the upper 350 m, particularly in the Pacific and Indian Oceans, shows robust features of decadal variations. During hiatus decades, the Indian Ocean shows an anomalous warming and accelerated OHC increase below 50 m, which is associated with a La Niña-like climate shift and an enhanced heat transport of the ITF. This Indian Ocean warming corresponds to a concurrent Pacific cooling in the 0–100 m layer, thus indicative of an Indo-Pacific heat rearrangement. We find that warming within 50–350 m mostly compensates the 0–50 m cooling in the Pacific and Indian Oceans so that enhanced deep-level (below 350 m) heat uptake is not required in these two basins. We therefore conclude that the ocean heat uptake in the upper 350 m in the Pacific and Indian Oceans is closely tied to the surface warming hiatus, whereas the regional pattern of deep ocean heat uptake (below 700 m) is not a valid basis to infer which basin drives the surface warming hiatus.

The vertical and interbasin heat redistribution in the ocean is not well understood. In addition to MOCs, the wind-driven decadal variability in ocean circulation is a plausible mechanism for heat redistribution^36^. For example, decadal variability of the Southern Ocean heat uptake is suggested to be caused by modulations in winds associated with the Southern Annual Mode^37^. The need to develop a rigorous energy view and test it for the hiatus^38^ spotlights the important problem of three-dimensional redistribution of heat in the ocean.

## Methods

### The OHC calculation

In this study, OHC within a certain layer is calculated as


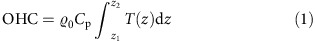


where 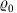 and *C*_P_ denote sea water density and specific heat capacity, respectively. *z*_1_ and *z*_2_ denote the upper and lower limits of the layer depth. *T*(*z*) denotes temperature profile, a function of depth *z*. Because we focus on the variability over the decadal time scales, a 12-month running is applied to the OHC time series. We calculate the OHC anomalies to the first year (year 1970) to manifest the continuous subsurface warming (Fig. 2), and to the period of 1970–2012 to reproduce the results by ref. 22 (Supplementary Fig. 9).

### The observational data sets

Four observational data sets are selected that cover the span of 1970–2012. They are composed of two categories: (1) objective analyses of *in-situ* observations (for example, expendable bathy-thermographs (XBTs), conductivity–temperature–depth (CTD) measurements from research ships and Argo floats) and (2) reanalysis products that assimilate a variety of available observations in an ocean model. The former includes the EN4 data^33^, the WOA data^34^ and the data from a research leading by Ishii^30^ (called the Ishii data in this study). The latter includes the ECMWF ORAS4 product^15^,^35^. In particular, the EN4 data is monthly mean temperature at 42 levels extending to beyond 5,000 m from 1900 to the present. The WOA data is annual mean temperature at 16 levels in the upper 700 m during 1955–2012. The Ishii data is monthly mean temperature at 24 levels in the upper 1,500 m during 1945–2012. The ECMWF ORAS4 product is monthly temperature at 42 levels extending to beyond 5,000 m during 1958–2014. For the Ishii data, the annual mean anomalies for globally integrated upper 700 m OHC during 1950–2014 is available from Japan Meteorological Agency. Due to a sparse sampling and its induced large OHC error in the early era (Supplementary Fig. 10), the period of 1970–2012 is selected for the SST and OHC analyses. In this study, to examine the changes in the OHC (Fig. 5) and temperature (Supplementary Fig. 5) between recent hiatus event and prior surge event, we consider the global and regional OHC (temperature) trends for the periods from 2003 to 2012 and from 1992 to 2001, and calculate the difference between the two periods.

### The CESM large ensemble simulations

The CESM community provides a production of a large ensemble (38 members) using a 1-degree CESM-CAM5 with biogeochemistry (BGC)^31^. Ensemble members go from 1920–2100 using historical forcing (1920–2005) and Representative Concentration Pathway 85 (RCP 8.5) forcing (2005–2100). We calculate the running 10-year linear trends of globally averaged SST from the 38-ensemble members and choose the members simulating the recent hiatus event by the criteria of negative decadal trend appearing within the period of 2002–2012. As shown in Supplementary Fig. 3, four members (M09, M16, M19 and M31) happen to capture the recent hiatus event, while four (M13, M29 M37 and M07) simulate a warming surge during the same span. Thus we collect the former four as the ‘Hiatus' group and the latter four as the ‘Surge' group. For each group, the ensemble mean SST and OHC are calculated and compared in Figs 1 and 2. The model drift is estimated from a 500-year pre-industrial control run and removed in the SST and OHC analyses. Moreover, to test the robustness of the OHC difference between the hiatus and surge events, we expand the Hiatus and Surge groups by including five pairs of hiatus and surge decades (Supplementary Table 1) in the future simulations and name the new groups are as the fHiatus and fSurge. To calculate the ensemble mean or standard deviation of the OHC (Figs 3 and 5) and temperature (Supplementary Fig. 5) of the fHiatus and fSurge, values in future hiatus and surge events are scaled to 2002–2011 by multiplying by a ratio of 38-ensmble mean global OHC trend during 2002–2012 to 38-ensmble mean global OHC trend during the event period.

## Additional information

**How to cite this article:** Liu, W. *et al.* Tracking ocean heat uptake during the surface warming hiatus. *Nat. Commun.* 7:10926 doi: 10.1038/ncomms10926 (2016).

## Supplementary Material

Supplementary Information Supplementary Figures 1-10 and Supplementary Table 1
