# Supplementary material for: Tracking ocean heat uptake during the surface warming hiatus
Source: Nat Commun. 2016 Mar 30;7:10926. doi: 10.1038/ncomms10926 (PMC4821003; doi:10.1038/ncomms10926)
Supplement: Supplementary Information — Supplementary Figures 1-10 and Supplementary Table 1 [file ncomms10926-s1.pdf]

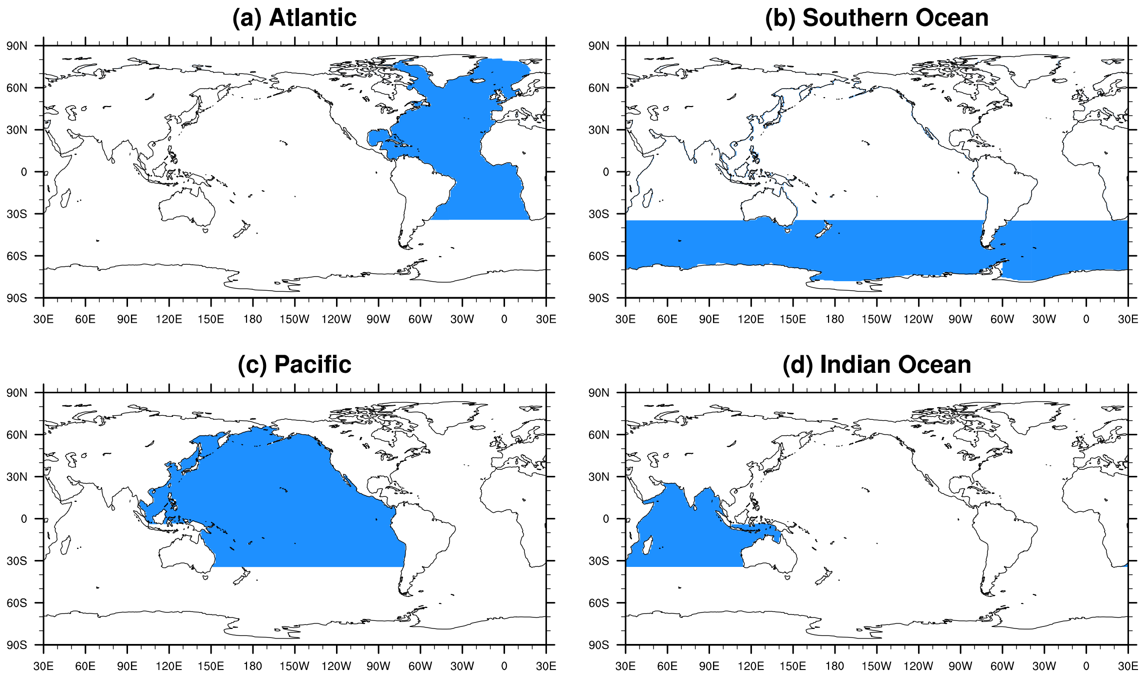

Supplementary Figure 1. The division of global oceans for (a) the Atlantic, (b) the Southern Ocean, (c) the Pacific and (d) the Indian Ocean.

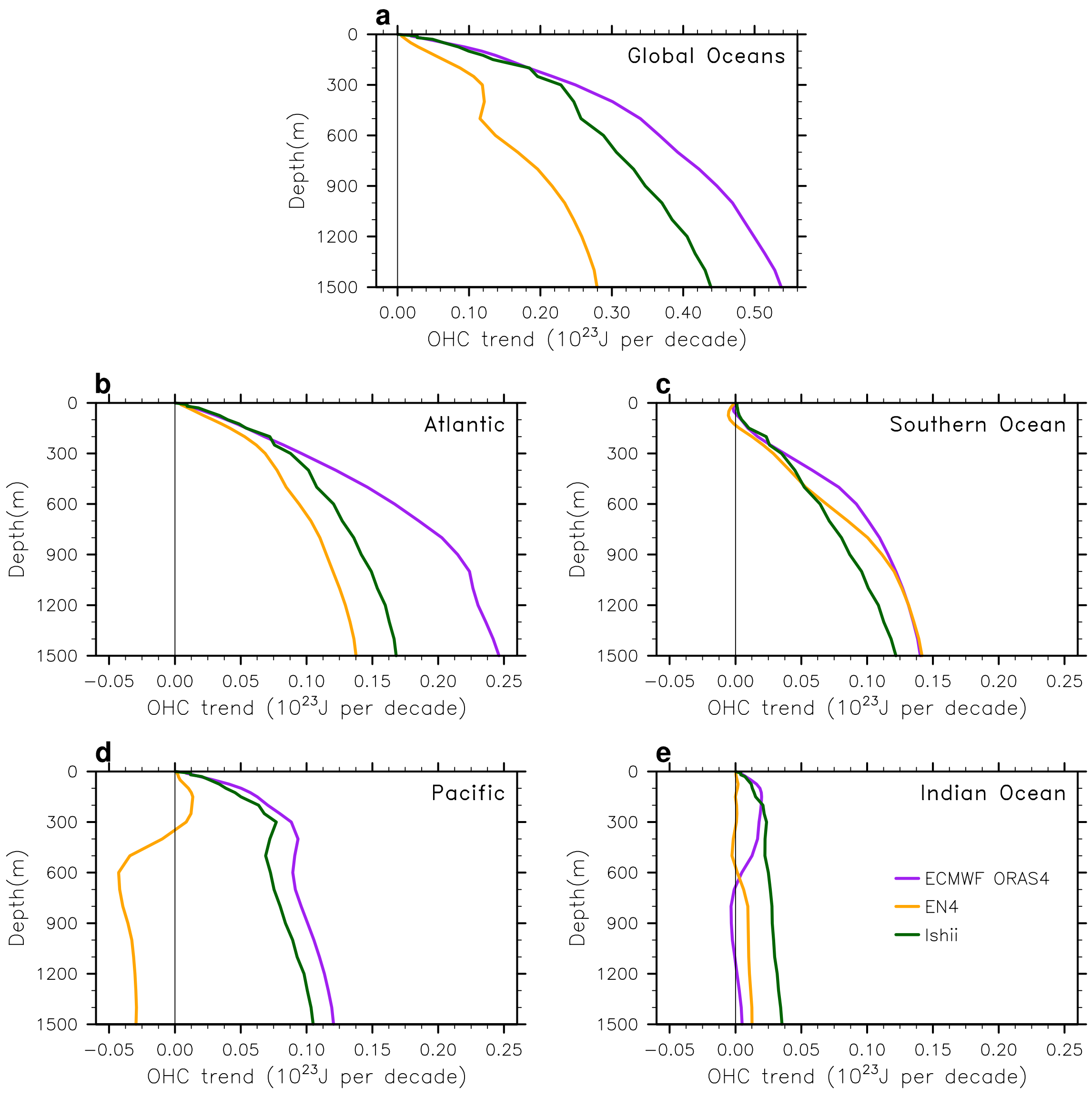

Supplementary Figure 2. Trends of OHC integrated from the surface to different depths in (a) global oceans, (b) the Atlantic, (c) the Southern Ocean, (d) the Pacific and (e) the Indian Ocean from observational datasets: the ECMWF ORAS4 reanalysis product (purple), the EN4 data (orange) and the Ishii data (dark green). Despite observational uncertainties, all three datasets show persistent heat storage into global deep oceans, since the positive trend monotonically increases when the global OHC is integrated over a deeper depth. Below 700m, most heat is sequestered in the Atlantic and the Southern Oceans.

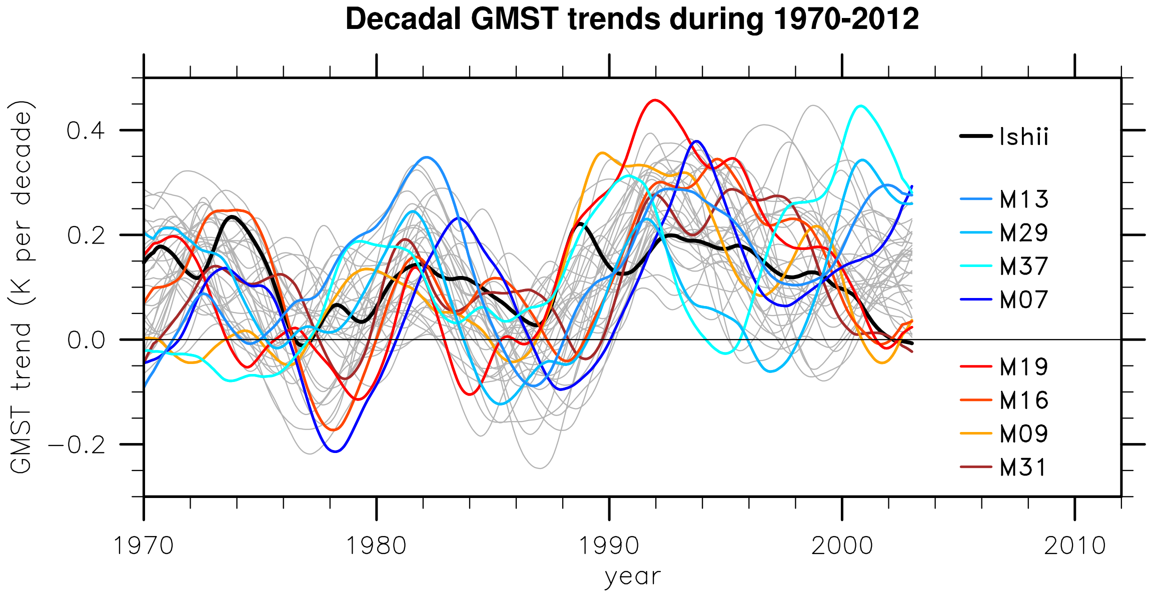

Supplementary Figure 3. The ten-year linear GMST trends from the Ishii data (black) and 38 CESM ensemble members. Four Hiatus members (M19, M16, M09 and M31) are colored in red, orange-red, orange and brown, which simulates the decadal GMST trend once negative during 2002-2012. Four Surge members (M13, M29 M37 and M07) are colored in dodger-blue, sky-blue, cyan and blue, which correspond to individual Hiatus members but with the largest warming trend during the same span. Other 30 members are colored in gray, simulating a moderate GMST warming during 2002-2012. A 12-month running mean is applied before the trend calculation.

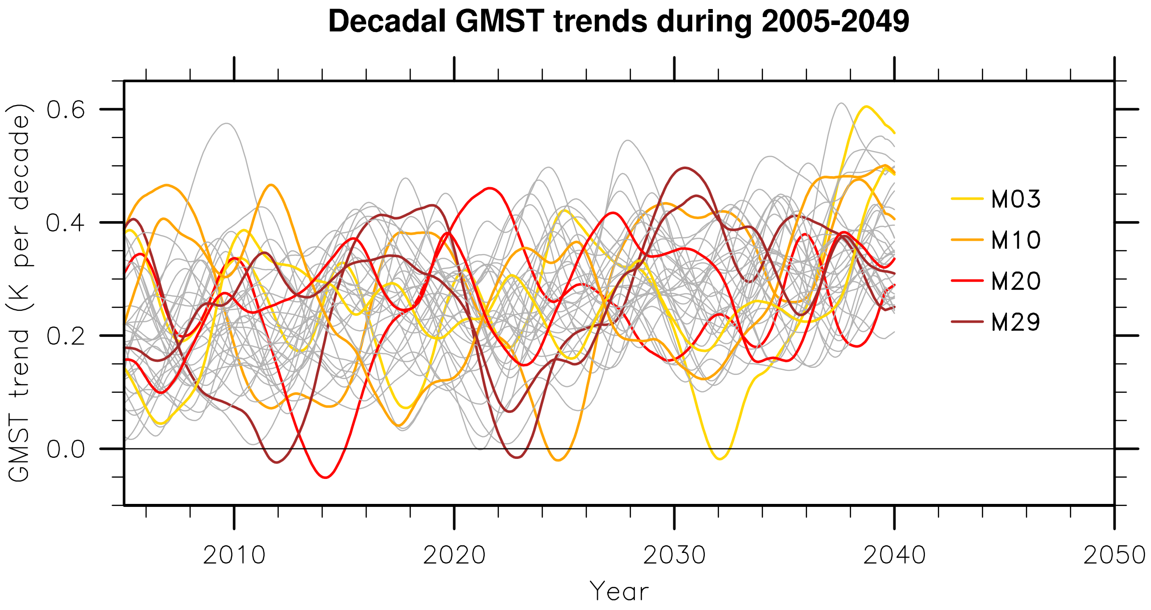

Supplementary Figure 4. The ten-year linear GMST trends in the CESM ensemble simulations during 2005-2049. Members simulating hiatus events (a negative decadal GMST trend) are highlighted by warm colors. The other members are colored in gray. A 12-month running mean is applied before the trend calculation.

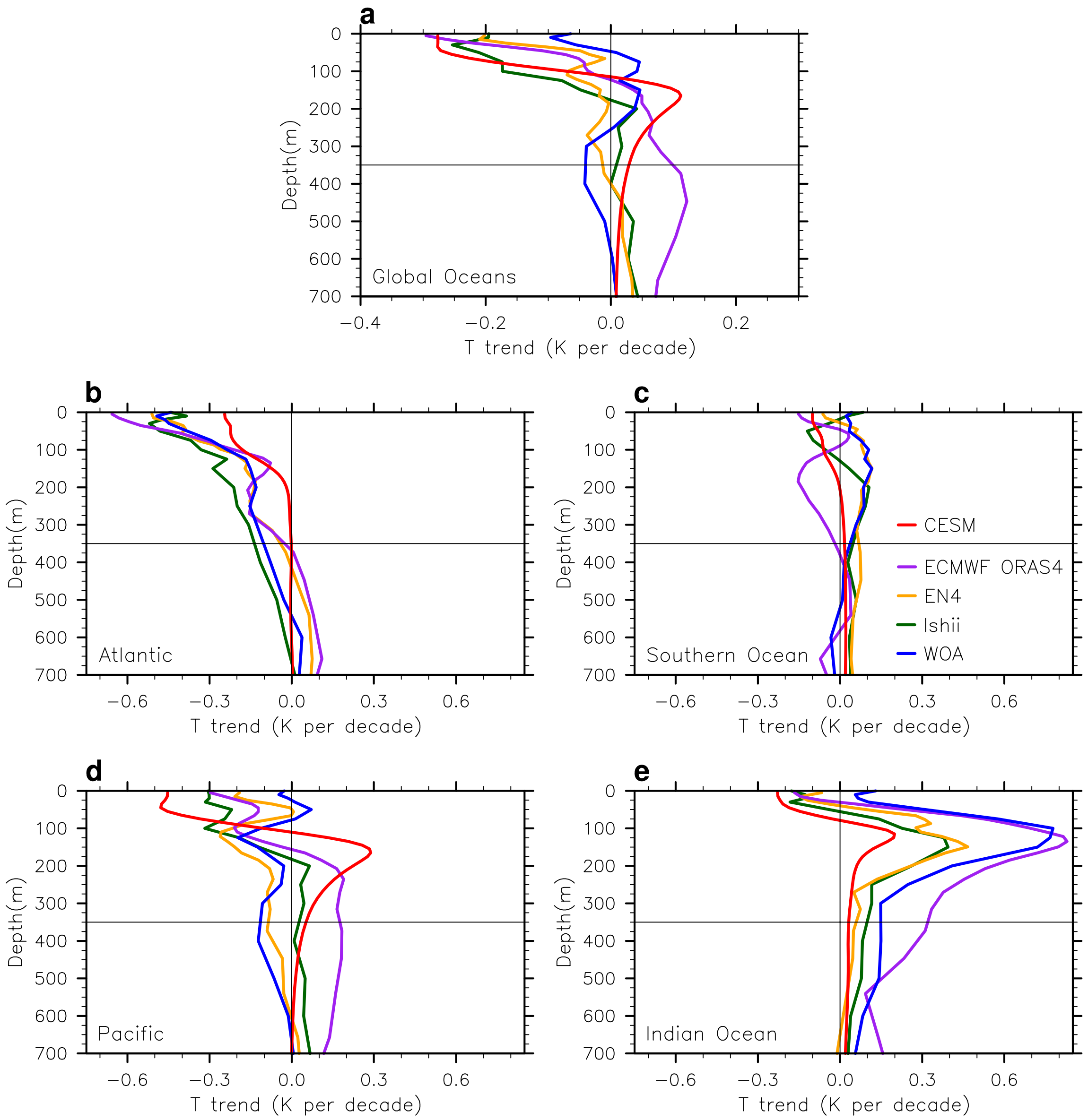

Supplementary Figure 5. Global and regional temperature trend differences as a function of depth between hiatus and surge events in (a) global oceans, (b) the Atlantic, (c) the Southern Ocean, (d) the Pacific and (e) the Indian Ocean from observational datasets and the CESM simulations. Observations include the ECMWF ORAS4 reanalysis product (purple), the EN4 data (orange), the Ishii data (dark green) and the WOA data (blue). The CESM result (red) shows the OHC trend difference between the fHiatus and fSurge groups (the fHiatus ensemble mean minus the fSurge ensemble mean).

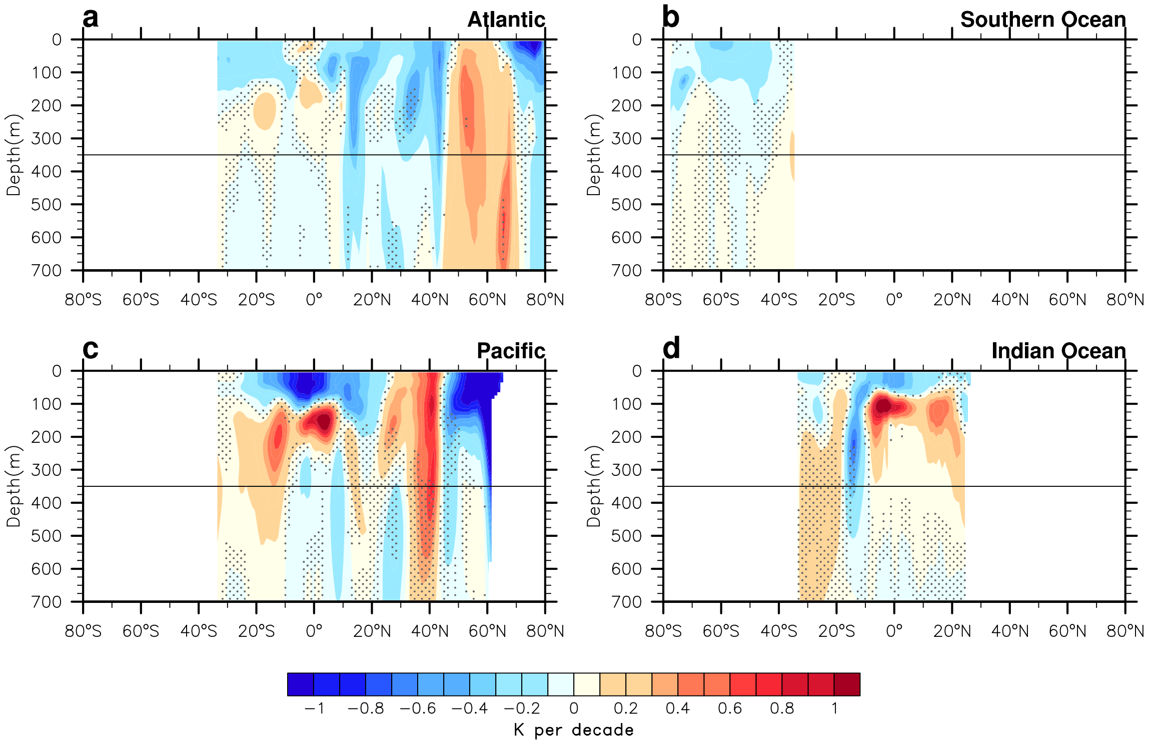

Supplementary Figure 6. The trend difference (the Hiatus ensemble mean minus the Surge ensemble mean) of zonal mean temperature (shading in *K* per decade) during 2002-2012 in (a) the Atlantic, (b) the Southern Ocean, (c) the Pacific and (d) the Indian Ocean. Stippling indicates region below 95% significance computed from a two-tailed *t* test.

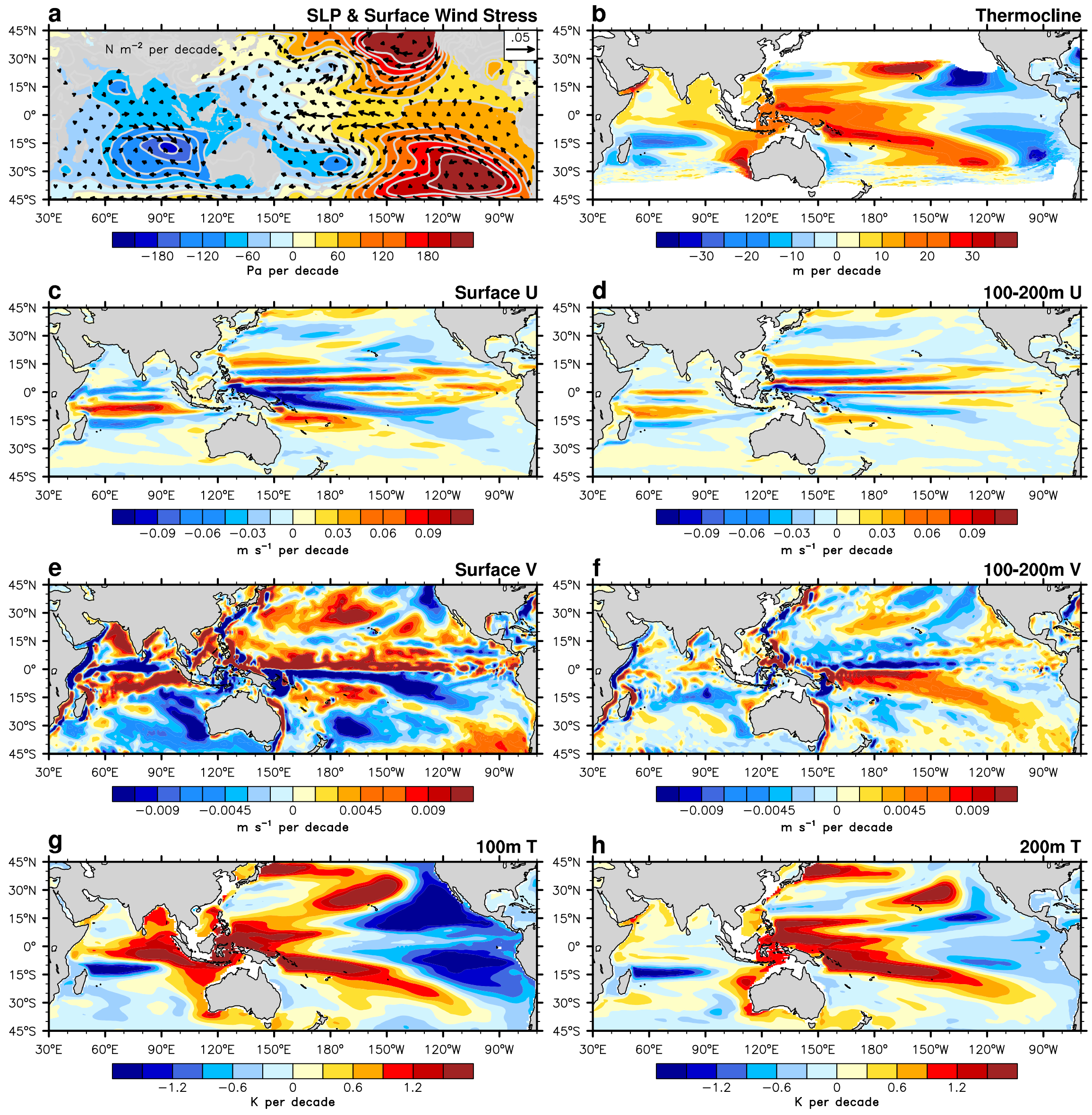

Supplementary Figure 7. The trend differences (the Hiatus ensemble mean minus the Surge ensemble mean) during 2002-2012 of (a) sea level pressure (SLP, shading in Pa per decade) and surface wind stress (vector, Nm^{-2} per decade), (b) thermocline depth that is defined as the depth of 20°C isotherm (shading in m per decade), (c) surface zonal velocity (shading in ms^{-1} per decade), (d) zonal velocity averaged over 100-200m (shading in ms^{-1} per decade), (e) surface meridional velocity (shading in ms^{-1} per decade), (f) meridional velocity averaged over 100-200m (shading in ms^{-1} per decade), (g) temperature at 100m (shading in K per decade) and (h) temperature at 200m (shading in K per decade).

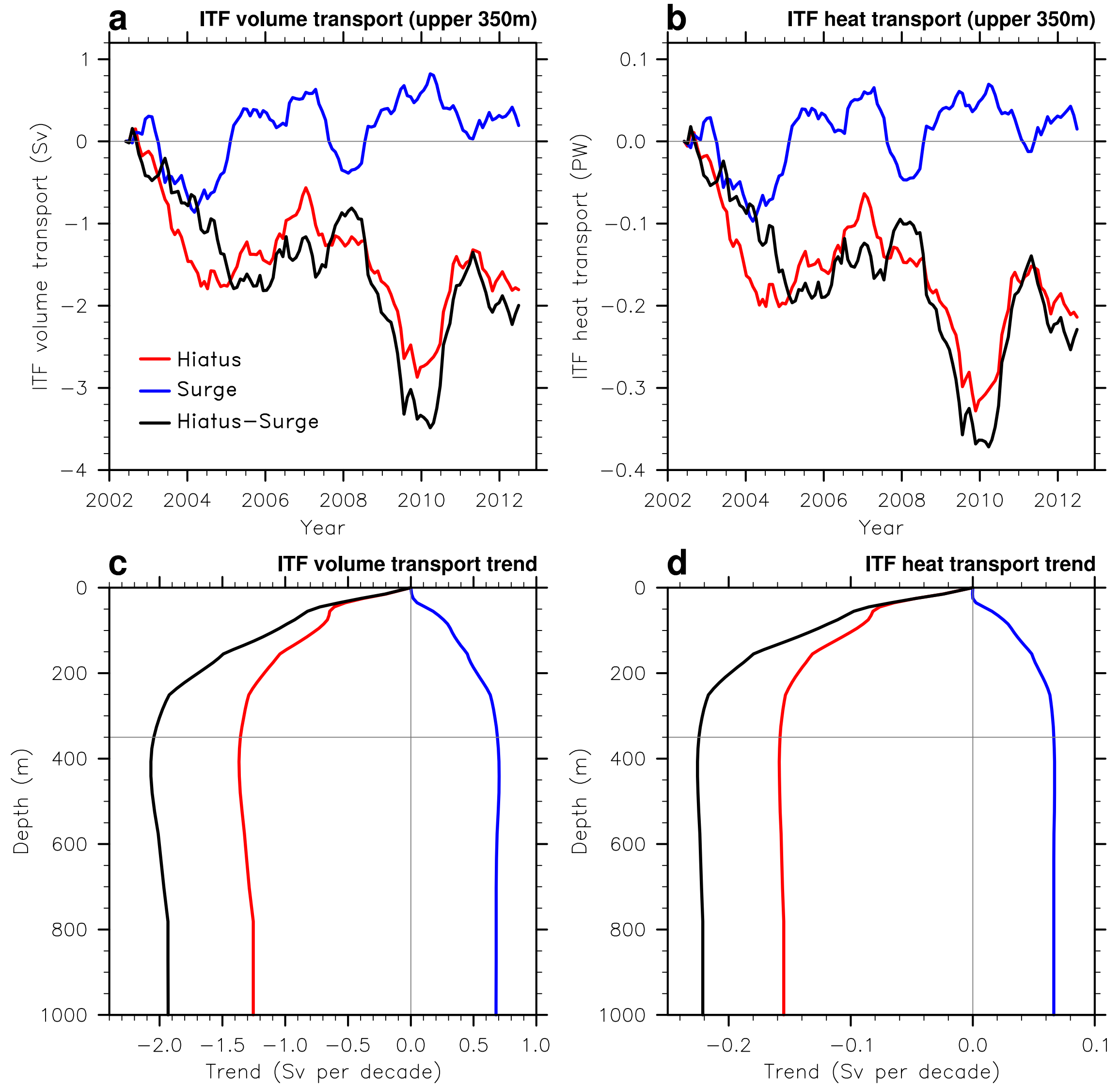

Supplementary Figure 8. Time series of the ITF (a) volume ($S_v, 1S_v = 1 \times 10^6 m^3 s^{-1}$) and (b) heat ($PW, 1PW = 1 \times 10^{15} W$) transports in the upper 350m during 2002-2012 for the Hiatus (red) and Surge (blue) ensemble means and the difference between the two (the Hiatus minus the Surge; black). All the curves are shown as a 12-month running mean by subtracting the annual mean value of the first year (year 2002). Panels (c) and (d) show trends of the ITF volume and heat transports during 2002-2012 as a function of depth in the upper 1000m for the Hiatus (red) and Surge (blue) ensemble means and the difference between the two (the Hiatus minus the Surge; black).

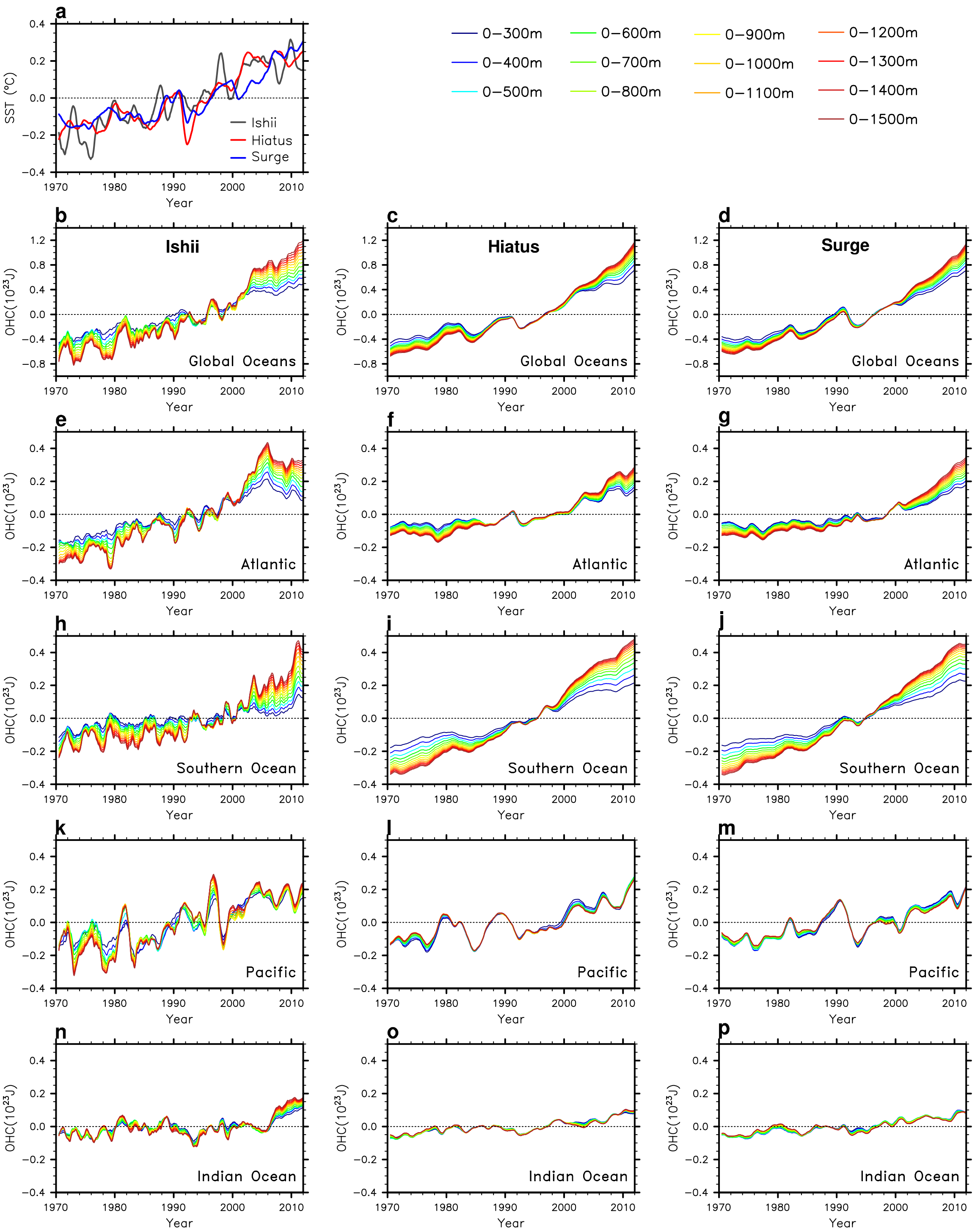

Supplementary Figure 9. (a) Monthly mean globally averaged SST from the Ishii data (black) and the Hiatus (red) and Surge (blue) ensemble means. (b-p) OHC integrated from the surface to indicated depths in global oceans, the Atlantic, Southern Ocean, Pacific and Indian Ocean from the Ishii data (panels b, e, h, k and n) and the Hiatus (panels c, f, i, l and o) and Surge (panels d, g, j, m and p) ensemble means. All the curves are shown as a 12-month running mean by subtracting the 1970-2012 mean.

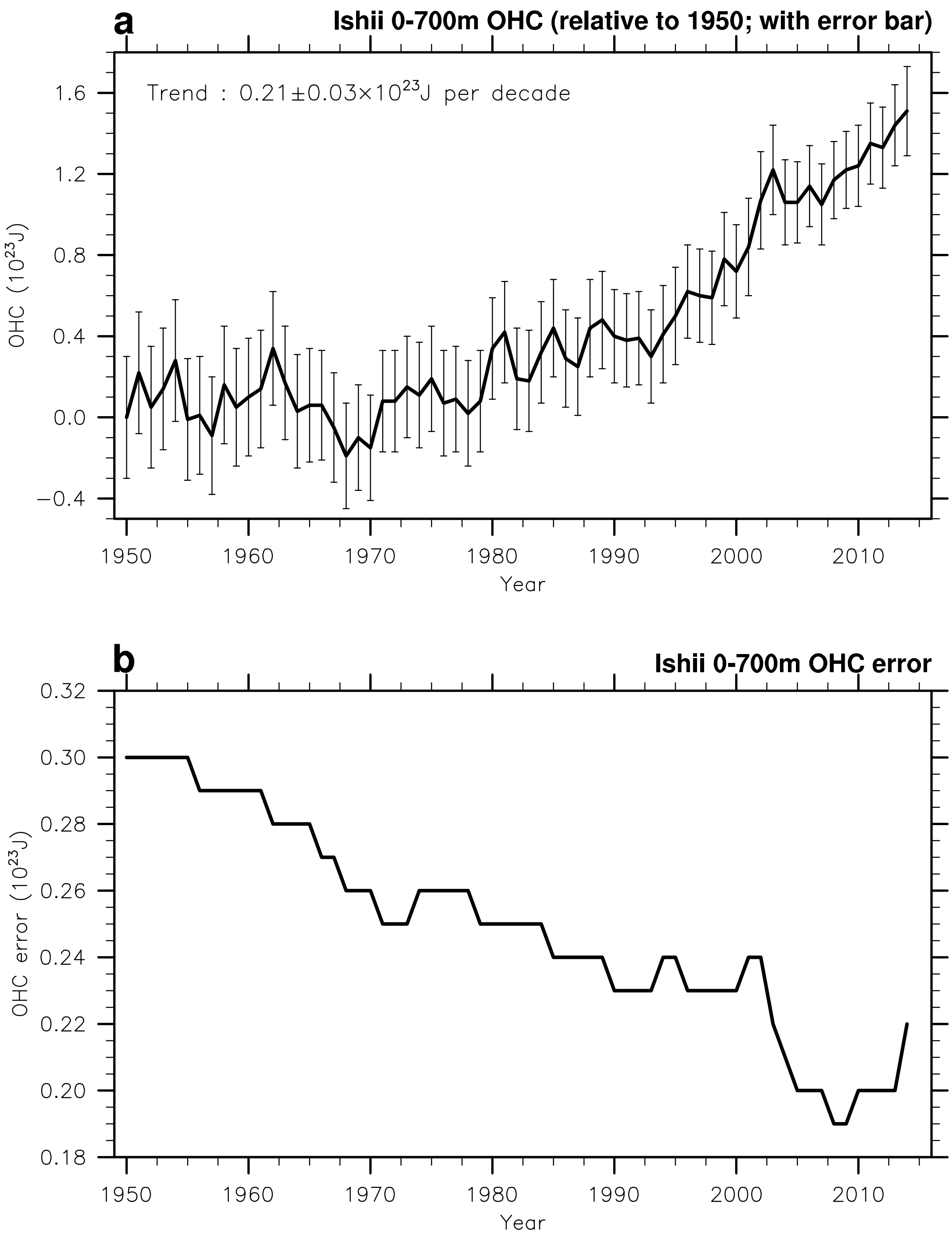

Supplementary Figure 10. (a) The annual mean of globally integrated upper 700m OHC during 1950-2014 (by subtracting the 1950 value). Error bar indicates a 95% confidence level. These data are obtained from Japan Meteorological Agency. (b) The time series of 0-700m global OHC errors during 1950-2014.

The hiatus and surge events in the 21st century

Event	Period	fHiatus	fSurge
1	2002-2011	M09	M13
2	2002-2011	M16	M29
3	2002-2011	M19	M37
4	2003-2012	M31	M07
5	2012-2021	M29	M11
6	2014-2021	M20	M14
7	2023-2032	M29	M20
8	2025-2034	M10	M35
9	2032-2041	M03	M10

Supplementary Table 1. Nine pairs of surface warming hiatus and surge events in the 21st century from a 38-member CESM large ensemble, which are grouped as the fHiatus and fSurge, respectively. Members simulating hiatus (surge) events are listed in the column of fHiatus (fSurge).
